# Melatonin Affects the Photosynthetic Performance of Pepper (*Capsicum annuum* L.) Seedlings under Cold Stress

**DOI:** 10.3390/antiox11122414

**Published:** 2022-12-07

**Authors:** Muhammad Ahsan Altaf, Huangying Shu, Yuanyuan Hao, Muhammad Ali Mumtaz, Xu Lu, Zhiwei Wang

**Affiliations:** 1 Key Laboratory for Quality Regulation of Tropical Horticultural Crops of Hainan Province, Sanya Nanfan Research Institute, Hainan University, Sanya 572025, China; 2 Key Laboratory for Quality Regulation of Tropical Horticultural Crops of Hainan Province, School of Horticulture, Hainan University, Haikou 570228, China; 3 Hainan Yazhou Bay Seed Laboratory, Sanya 572025, China

**Keywords:** photosynthesis, pigment molecules, cold stress, pepper, melatonin

## Abstract

Photosynthesis is an important plant metabolic mechanism that improves carbon absorption and crop yield. Photosynthetic efficiency is greatly hampered by cold stress (CS). Melatonin (ME) is a new plant growth regulator that regulates a wide range of abiotic stress responses. However, the molecular mechanism of ME-mediated photosynthetic regulation in cold-stressed plants is not well understood. Our findings suggest that under low-temperature stress (15/5 °C for 7 days), spraying the plant with ME (200 µM) enhanced gas exchange characteristics and the photosynthetic pigment content of pepper seedlings, as well as upregulated their biosynthetic gene expression. Melatonin increased the activity of photosynthetic enzymes (Rubisco and fructose-1, 6-bisphosphatase) while also enhancing starch, sucrose, soluble sugar, and glucose content under CS conditions. Low-temperature stress significantly decreased the photochemical activity of photosystem II (PSII) and photosystem I (PSI), specifically their maximum quantum efficiency PSII (Fv/Fm) and PSI (Pm). In contrast, ME treatment improved the photochemical activity of PSII and PSI. Furthermore, CS dramatically reduced the actual PSII efficiency (ΦPSII), electron transport rate (ETR) and photochemical quenching coefficient (qP), while enhancing nonphotochemical quenching (NPQ); however, ME treatment substantially mitigated the effects of CS. Our results clearly show the probable function of ME treatment in mitigating the effects of CS by maintaining photosynthetic performance, which might be beneficial when screening genotypes for CS tolerance.

## 1. Introduction

Plants face adverse environmental conditions, including temperature fluctuations (low and high), drought, salinity, variations in nutrient availability, soil, and air pollution, as well as floods [1]. Climate change has a significant impact on crop biochemistry and the quantity and quality of agricultural produce [2]. Plants respond to environmental stresses in a variety of ways, including through morphological, anatomical, physiological, biochemical, molecular, and metabolic alterations [3]. Temperature variations (low and high) have been recognized as a critical constraint for plant growth and productivity. Cold stress (CS) restricts natural plant dispersal and reduces agricultural production [4].

Photosynthesis is one of the main physiological processes that are sensitive to CS, which may lead to a reduction in growth and yield in plants at low temperatures [5]. Plants exposed to low temperatures have lower photosynthetic efficiency and lower levels of photosynthetic pigments in their leaves [6]. Low temperatures rapidly decrease the water content of leaves and enhance the viscosity of their membranes [7]. Temperature stress (low and high) also negatively affects photosynthetic systems [8]. Rubisco is one of the photosynthetic enzymes that are sensitive to low temperatures and which have a direct role in CO_2_ fixation [9]. Furthermore, CS directly impacts the performance of photosystems and photosynthetic machinery [10]. CS has a detrimental effect on the structure of thylakoids, chlorophyll content, the activity of photosynthetic enzymes, and photosynthetic electron transport (PET) [11]. Chlorophyll production is impaired when plants are subjected to CS [1]. The efficiency of PET in plants is dramatically reduced under CS, resulting in excessive energy production, and triggering photoinhibition [12]. Jiang et al. [13] revealed that CS has a direct effect on the photosynthetic apparatus, primarily through the induction of photoinhibition at photosystem I (PSI) and photosystem II (PSII). The photoinhibition of PSI and PSII occurs under CS [14]. PSI is more vulnerable to cold than PSII, as the recovery of PSI photoinhibition after a cold period is substantially slower than that of PSII [15,16]. It was previously reported that PSII photoinhibition was exacerbated by CS, which reduced the replenishing of D1 proteins [17].

Bio-stimulants are considered an environmentally friendly and effective method of improving plant stress resistance [18]. Previous research indicates that stress mechanisms are mediated by the signaling and associated activities of plant growth regulators (PGRs). ME is one of these versatile and naturally powerful antioxidants that have a broad spectrum of applications, and is found in both animals and plants [19]. ME is associated with several developmental processes, from seed germination to fruit maturation [20]. ME has been documented to be involved in plant defense systems in stressful conditions, such as CS [12,21,22,23]. ME application promotes the photosynthetic apparatus in many plant species under CS conditions [24,25,26]. Zuo et al. [27] revealed that under nano-ZnO stress, ME application remarkably enhanced chlorophyll content, improved Rubisco and ATPase activities, and increased photosynthetic C assimilation and energy transport efficiency in wheat. ME efficiently enhanced pigment molecules, carbon assimilation, the activity of Rubisco and fructose-1, 6-bisphosphatase, chlorophyll content, and upregulated the expression of photosynthetic related genes in tomato [20,28]. Exogenous ME supplementation significantly improved the photosynthetic capacity in tomato seedlings under CS. Furthermore, at LTs, ME pre-treated plants showed higher electron transfer rates and quantum yields of PSI and PSII [25]. In another study, ME application enhanced the photosynthetic performance of melon seedlings under CS conditions [26]. In a recent study, Siddiqui et al. [29] reported that ME effectively increased pigment content, carbon flow in the Calvin cycle, Rubisco, FBPase, and SBPase activity in tomatoes under cadmium stress, whereas reduced chlorophyllase activity was also found to have occurred. Additionally, exogenous ME also protects the whole photosystem from a wide range of adverse environments [30,31]. Nonetheless, there is insufficient information available that exogenous ME plays a crucial role in the photosynthetic responses of plants to CS. Thus, it is critical to understand how ME modulates photosynthetic efficacy in cold-stressed pepper plants.

In the Solanaceae family, pepper (Capsicum annuum) is one of the most widely consumed horticultural crops around the globe [32]. Pepper crops are native to the tropics and, therefore, their optimal growth temperature is between 25 and 30 °C. Temperature fluctuations influence a wide range of physiological activities and morphological development. Pepper growth is inhibited when the temperature falls below 15 °C [33] and pepper plants are highly sensitive to cold temperatures, while their growth and development are significantly damaged under severely low winter temperatures [34,35]. The present study investigated the probable role of exogenous ME on the photosynthetic apparatus and related gene expression in the leaves of pepper seedlings during CS.

## 2. Materials and Methods

### 2.1. Experimental Material and Setup

Pepper seeds (*Capsicum annuum* L. Var. Ca-59, collected from the vegetable germplasm bank of Hainan University) were sown in seedling trays filled with nutrient-rich peat soil. After five weeks of sowing, uniform-sized seedlings were transferred to a plastic container filled with nutrient-rich peat soil. Seedlings were divided into three treatments after a five-day adaptation period. The treatments were as follows: (1) control (CK); cold stress (CS); melatonin plus cold stress (ME + CS). For the ME + CS treatment, for four days, the plants were sprayed (50 mL) with 200 µM L−1 ME once a day at 6:00 p.m., whereas the control plants were treated with distilled water. CS was given by shifting the plants into a controlled growth chamber with a temperature of 15/5 °C, a light/dark period of 12 h/12 h, a photon flux density of 100 µmol m^−2^ s ^−1^, and a relative humidity of 80%. The ME (200 µM) dose was selected based on prior research [36]. After 7 days of stress treatment, plant samples were taken and kept at −80 °C for further analysis. Each treatment consisted of three replications, with 15 plants per replication.

### 2.2. Photosynthetic Pigments and SPAD Index

The chlorophyll and carotenoid contents of the leaves were analyzed according to the method of Arnon [37]. In brief, 0.5 g of fresh leaves was ground in 10 mL of 80% acetone, followed by centrifugation of the homogenate at 5000× *g* for 10 min, and a spectrophotometer was used to measure the photosynthetic pigment. Images of autofluorescence emitted at 650–700 nm (chlorophylls) or 500–550 nm (carotenoids) were obtained after excitation with the 488 nm ray line of an argon laser. To observe the chlorophyll and carotenoid (lycopene) contents, a TCS SP2 laser confocal microscope (Leica, Heidelberg, Germany) was used, as previously reported by Mumtaz et al. [38].

The SPAD-502 (Konica Minolta, Camera, Ltd., Tokyo, Japan) measures the leaf absorbance, in both red and near-infrared regions, in order to determine the chlorophyll concentration. Two LEDs with peak wavelengths of 650 nm and 940 nm emit light with the measuring head closed, and the sequence runs from an emitting window to a photodiode detector. In the measuring head, during the passing of light through the leaf sample, a certain amount strikes the receptor after being transmitted through the leaf, which is then converted into an electrical signal. The SPAD values are then calculated by the SPAD502 by dividing the light transmission intensities at 650 nm by those at 940 nm. These calculated SPAD values then exhibit the relative content of chlorophyll within the sample leaves [39].

### 2.3. Leaf Gas Exchange Parameters and Scanning Electron Microscopy (SEM)

A portable photosynthesis system (CIRAS-3, Hansatech Co., Amesbury, MA, USA) was used to measure the leaf gas exchange parameters (net photosynthetic rate (Pn), stomatal conductance (Gs), intercellular CO_2_ concentration (Ci), and transpiration rate (Tr)). Fully developed leaves were used to calculate the gas exchange parameters [40].

To prepare the leaf samples for SEM, they were rapidly treated with 2.5% glutaraldehyde and then with 1% OsO4 in (0.1 M) phosphate-buffered saline (PBS; pH 6.8) to avoid any damage. A graded ethanol solution was used to dry the treated leaves; then, they were transferred to an alcohol + iso-amyl acetate (1:1, *v*/*v*) combination, and finally to pure iso-amyl acetate. Finally, the samples were vacuum-dried with liquid CO_2_ in a Hitachi Model HCP-2 and coated with gold–palladium in a Hitachi Model E-1010 ion sputter. The SEM observations were made with an S-4800 microscope (Model TM-1000, Hitachi Led., Tokyo, Japan).

### 2.4. Chlorophyll a Fluorescence Characteristics Measurement

The chlorophyll a fluorescence induction kinetics were measured using Dual portable fluorescence (Dual-PAM-100, Walz, GmBH Eichenring, Effeltrich, Germany); various parameters were measured using Dual-PAM software. Chlorophyll fluorescence and P700+ absorbance changes were utilized to assess the photochemical activity of the photosystems PSII and PSI. To determine the parameters of the photosynthetic electron transport chain, a previously established approach was used with the Handy Plant Efficiency Analyzer (Handy PEA, Hanstech, Norfolk, UK) [20].

### 2.5. Starch and Sucrose Assays

Dry leaf samples were homogenized in ethanol and then incubated in a water bath at 80 °C for 30 min to measure the starch and sugar contents. The top aliquot was utilized for the sugar analysis [41]. The supernatant was extracted from the residue using 10 mL of 52% HClO_4_ (*v*/*v*) and was used to determine the starch content [42].

### 2.6. FBPase and Rubisco Activity

The activity of Rubisco and FBPase was recorded according to the procedure quoted by Jahan et al. [20]. For the Rubisco activity, 0.3 g of composite leaves was ground in 2 mL of 1% polyvinylpyrrolidone. The homogenate mixture was centrifuged at 4 °C for 15 min at 15,000× *g*, and the obtained supernatant was employed to assess the enzyme activity. For the FBPase activity, after 7 days of CS, the composite leaf tissues of the pepper were obtained and crushed into a fine powder in liquid nitrogen using a precooled mortar and pestle. The reaction mixture was used to homogenize the powdered leaf tissues. The homogenate was centrifuged at 15,000× *g* for 10 min, and the supernatant was utilized to perform the FBPase activity analysis.

### 2.7. Gene Expression through Quantitative Real-Time PCR

After 7 days of CS treatment, total RNA was extracted from individual treatment leaf samples using Trizole reagent, following the manufacturer’s instructions. The quality and purity of the extracted RNA was examined using agarose gel electrophoresis, with a Nanodrop 2000 spectrophotometer^®^ (Thermo, Deutshland, Germany). For the complementary DNA (cDNA) synthesis, extracted RNA was reverse-transcribed using the Vazyme HiScript II QRT SuperMix for qPCR (+gDNA wiper) 1st-strand cDNA synthesis kit (Vazyme, Nanjing, China), following the manufacturer’s protocol, and the cDNA was used as a template for the quantitative real-time PCR (qRT PCR) analysis. The qRT PCR was performed with 96-well plates using an Mx3000 P qPCR system (Agilent Technologies, Santa Clara, CA, USA) and the Roche FastStart Essential DNA Green Master kit (Roche, Indianapolis, IN, USA). The primers used in this experiment are given in Table S1. The actin gene was used as an internal reference gene. The values of relative expression level changes were calculated using the formula 2^−ΔΔCt^ [43].

### 2.8. Statistical Analysis

Statistical Software SPSS (IBM SPSS 22.0, IBM Corporation, New York, NY, USA) was used to perform the analysis. Duncan’s multiple range test (DMRT) (*p* < 0.05) was employed to analyze the differences between treatments. The means ± SEs of three biological replicates were used to present the data.

## 3. Results

### 3.1. This Photosynthetic Pigments, Carotenoids, and SPAD Index (Relative Chlorophyll Content)

**Figure 1:**
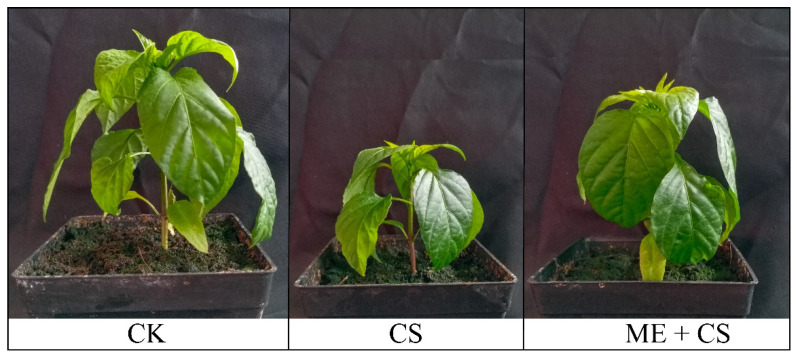
Visual demonstration of pepper seedlings under ME and CS conditions.

**Figure 2:**
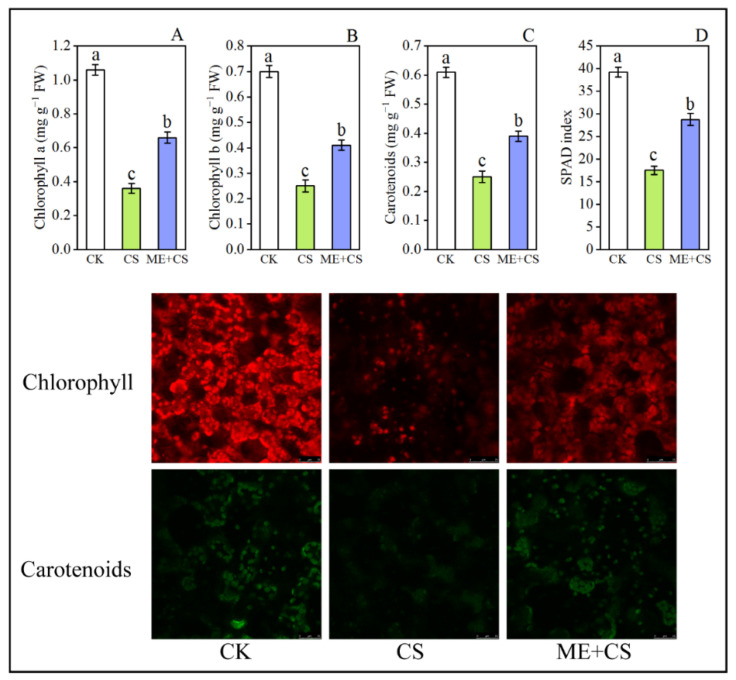
Effect of foliar application of melatonin on chlorophyll a (**A**), chlorophyll b (**B**), carotenoid content (**C**), and SPAD index (**D**) of pepper seedlings subjected to cold stress. Image of the pepper leaves’ chlorophyll and carotenoid (lycopene) auto-fluorescence. Plastids containing chlorophylls appear red and those containing carotenoids appear green. Scale bars: 10 μm. The results are means ± standard errors for n = 9. Significant differences are exhibited by lowercase letters (*p* ≤ 0.05), according to Duncan’s multiple range test.

The phenotypic appearance of the leaves showed that the pigment content declined dramatically when the plant was stressed by CS (Figure 1). After CS, the chlorophyll a, chlorophyll b, and carotenoid contents of the leaves were reduced to 66%, 64%, and 59% of the control, respectively. In ME-treated seedlings, the reduction in these parameters was considerably lower, with 37%, 41%, and 36%, respectively, of the initial levels remaining after CS (Figure 2A–C). The SPAD index decreased significantly in cold-stressed seedlings. Contrarily, under CS conditions, ME application efficiently increased the SPAD values in pepper leaves (Figure 2D).

### 3.2. Pigments-Related Genes Expression

**Figure 3:**
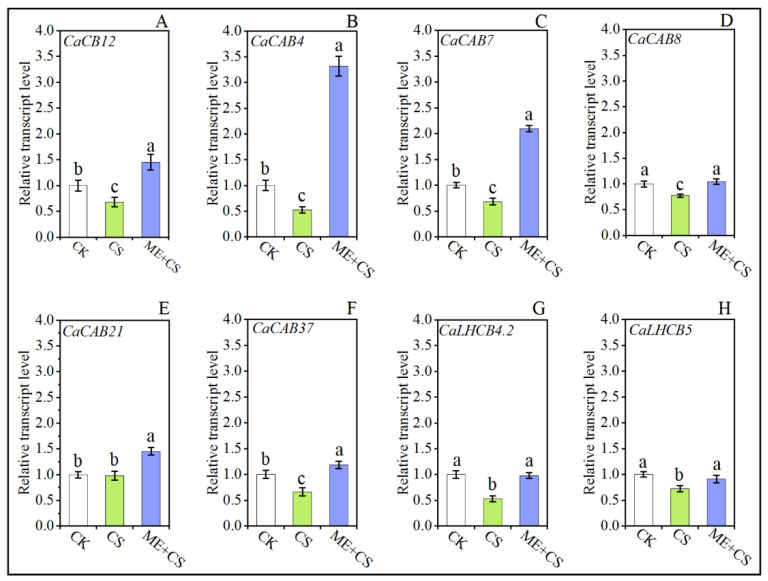
Combined effects of melatonin treatment and cold stress over 7 days on chlorophyll-related gene (*CaCB12* (**A**), *CaCAB4* (**B**), *CaCAB7* (**C**), *CaCAB8* (**D**), *CaCAB21* (**E**), *CaCAB37* (**F**), *CaLHCB4.2* (**G**), *CaLHCB5* (**H**)) expression in pepper seedlings. The results are means ± standard errors for n = 9. Significant differences are exhibited by lowercase letters (*p* ≤ 0.05), according to Duncan’s multiple range test.

Figure 2 shows the transcript abundance of chlorophyll a/b-binding protein (*CaCB12*), chlorophyll a/b-binding protein 4 (*CaCAB4*), chlorophyll a/b-binding protein 7 (*CaCAB7*), chlorophyll a/b-binding protein 8 (*CaCAB8*), chlorophyll a/b-binding proteins 21 (*CaCAB21*), chlorophyll a/b-binding protein 37 (*CaCAB37*), chlorophyll a/b-binding protein CP29.2 (*CaLHCB4.2*), and chlorophyll a/b-binding protein CP26 (*CaLHCB5*) under CK, CS, and ME + CS. Chlorophyll a/b-binding proteins are recognized as crucial components of light-harvesting complex II. After CS, the transcript levels of the chlorophyll-related genes *CaCB12*, *CaCAB4*, *CaCAB7*, *CaCAB8*, *CaCAB21*, *CaCAB37*, *CaLHCB4.2*, and *CaLHCB5* were dramatically reduced to 32%, 47%, 31%, 22%, 76%, 33%, 47%, and 27% of the control, respectively. The transcript levels of genes *CaCB12*, *CaCAB4*, *CaCAB7*, *CaCAB8*, *CaCAB21*, and *CaCAB37* were higher after ME + CS than in the control. Furthermore, following ME + CS treatment, the reduction in *CaLHCB4.2* and *CaLHCB5* gene expression levels was considerably lower than the initial levels remaining after CS (Figure 3). The current findings reveal that the treatment of leaves with ME effectively enhances the chlorophyll content of pepper seedlings subjected to cold stress.

### 3.3. Leaf Exchange Traits and Stomatal Opening

**Figure 4:**
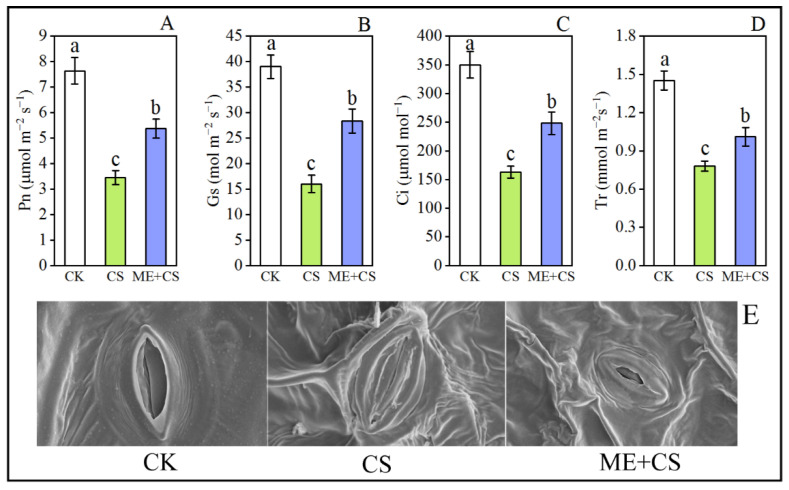
Combined effects of melatonin treatment and cold stress over 7 days on gas exchange parameters (Pn (**A**), Gs (**B**), Ci (**C**), and Tr (**D**)) and stomatal opening (**E**) in pepper seedlings. The results are means ± standard errors for n = 9. Significant differences are exhibited by lowercase letters (*p* ≤ 0.05), according to Duncan’s multiple range test.

We evaluated the gas exchange properties of plants with various treatments to examine the effect of ME on their photosynthetic capability. The leaf gas exchange traits, such as those of Pn, Gs, Ci, and Tr, were considerably reduced under CS conditions (Figure 4A–D). After CS, the Pn, Gs, Ci, and Tr values of the leaves were decreased to 54%, 58%, 53%, and 46% of the control, respectively (Figure 4A–D). In ME-treated seedlings, the decline in these parameters was noticeably lower, with 29%, 27%, 29%, and 30%, respectively, of the initial levels remaining after CS (Figure 4A–D). Furthermore, SEM demonstrated that the supplementation of exogenous melatonin might have altered the opening of stomata in response to CS (Figure 4E). ME may promote stomatal opening by osmotically holding water in the leaves.

### 3.4. Chlorophyll Fluorescence Traits

**Figure 5:**
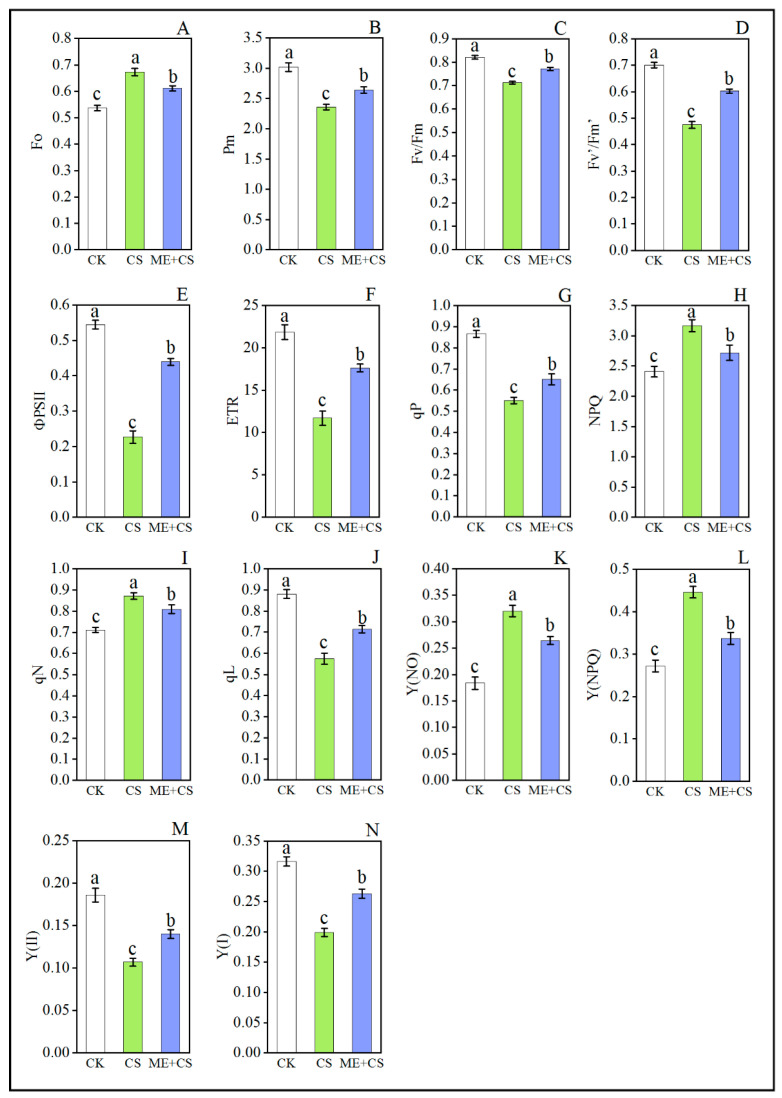
Combined effects of melatonin treatment and cold stress over 7 days on chlorophyll fluorescence (Fo (**A**), Pm (**B**), Fv/Fm (**C**), Fv’/Fm’ (**D**), фPSII (**E**), ETR (**F**), qP (**G**), NPQ (**H**), qN (**I**), qL (**J**), Y(NO) (**K**), Y(NPQ) (**L**), Y (II) (**M**) and Y (I) (**N**)) traits in pepper seedlings. The results are means ± standard errors for n = 9. Significant differences are exhibited by lowercase letters (*p* ≤ 0.05), according to Duncan’s multiple range test.

Cold stress significantly reduced the maximal chlorophyll fluorescence after full dark adaptation (Pm), maximum photochemical efficiency under dark adaptation (Fv/Fm), PSII maximum efficiency under light (Fv’/Fm’), actual photosynthetic efficiency (фPSII), photosynthetic electron transfer rate (ETR), photochemical quenching (qP), and coefficient of photochemical quenching (qL), compared with the CK treatment. Meanwhile, the initial chlorophyll fluorescence yield (Fo) increased under CS along with nonphotochemical fluorescence quenching (NPQ), and the coefficient of nonphotochemical fluorescence quenching (qN), compared with the CK group (Figure 5A–L). For instance, CS considerably decreased the Pm, Fv/Fm, Fv’/Fm’, фPSII, ETR, qP, and qL values by 21%, 13%, 32%, 58%, 46%, 36%, and 34%, respectively, and increased the Fo, NPQ, and qN values by 25%, 31%, and 22%, respectively, suggesting that thermal energy dissipation was increased in PSII. Nevertheless, the values of Pm, Fv/Fm, Fv’/Fm’, фPSII, ETR, qP, and qL increased while the values of Fo, NPQ, and qN reduced with ME application in pepper plants subjected to CS conditions (Figure 5A–L).

Under CS conditions, the effective quantum efficiencies of PSII (Y (II)) and PSI (Y (I)) were reduced to 42% and 37%, respectively, compared with CK plants. In contrast, when pepper seedlings were pretreated with ME, these traits only declined by 24% and 16%, respectively, when compared with the CK group (Figure 5M,N). The Y (II) value in pepper leaves was reduced after exposure to CS, whereas the values of nonregulated energy dissipation (Y (NO)) and regulated energy dissipation (Y (NPQ)) were significantly enhanced compared with other treatments. Conversely, the Y (NO) and Y (NPQ) values in pepper plants pretreated with ME and CS were reduced to 17% and 24%, respectively, when compared to the CS treatment (Figure 5K,L). The current findings show that ME helps plants to balance the absorption of light energy, thereby protecting the photosynthetic apparatus.

### 3.5. Photosynthesis-Related Gene Expression

**Figure 6:**
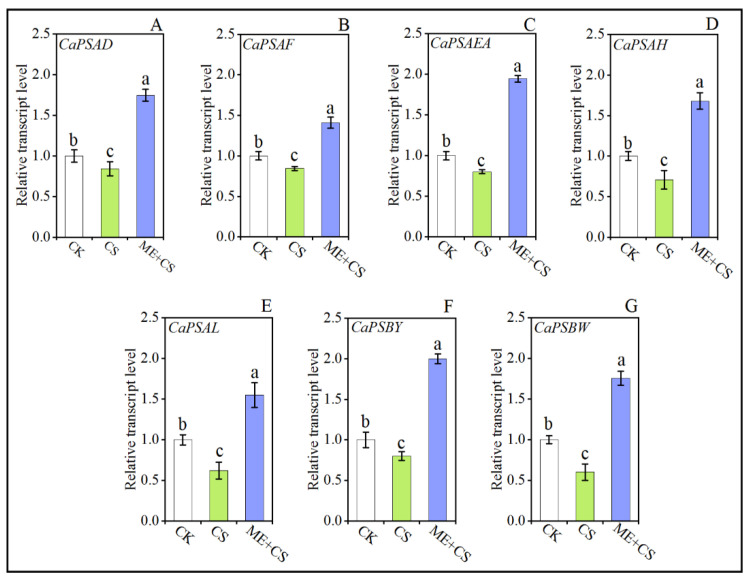
Combined effects of melatonin treatment and cold stress on photosynthesis-related gene (*CaPSAD* (**A**), *CaPSAF* (**B**), *CaPSAEA* (**C**), *CaPSAH* (**D**), *CaPSAL* (**E**), *CaPSBY* (**F**), *CaPSBW* (**G**)) expression in pepper seedlings. The results are means ± standard errors for n = 9. Significant differences are exhibited by lowercase letters (*p* ≤ 0.05), according to Duncan’s multiple range test.

To study the possible role of ME in terms of photosynthesis-related gene expression, we assessed the expression of many genes associated with both the PSI reaction center (Photosystem I reaction center subunit II (*CaPSAD*), Photosystem I reaction center subunit III (*CaPSAF*), Photosystem I reaction center subunit IV A (*CaPSAEA*), Photosystem I reaction center subunit VI (*CaPSAH*), and Photosystem I reaction center subunit XI (*CaPSAL*)) and the PSII reaction center (Photosystem II core complex proteins psbY (*PSBY*) and Photosystem II reaction center W protein (*PSBW*)). After CS, the transcript levels of the photosynthesis-related genes *CaPSAD*, *CaPSAF*, *CaPSAEA*, *CaPSAH*, *CaPSAL*, *CaPSBY*, and *CaPSBW* were dramatically reduced to 15%, 16%, 19%, 29%, 37%, 19%, and 39%, of the control, respectively. The transcript levels of the *CaPSAD*, *CaPSAF*, *CaPSAEA*, *CaPSAH*, *CaPSAL*, *CaPSBY*, and *CaPSBW* genes were higher after ME + CS than in the control (Figure 6). These results suggest that ME modulates the efficiency of photosynthesis by regulating the expression of genes involved in photosynthesis.

### 3.6. FBPase and Rubisco Enzyme Activity

**Figure 7:**
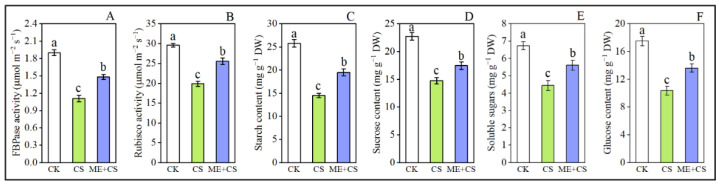
Effects of melatonin treatment on the FBPase (**A**) Rubisco (**B**) starch (**C**), sucrose (**D**), soluble sugars (**E**), and glucose (**F**) contents of pepper seedlings subjected to cold stress. The results are means ± standard errors for n = 9. Significant differences are exhibited by lowercase letters (*p* ≤ 0.05), according to Duncan’s multiple range test.

In order to better understand the ME-mediated photosynthesis process, we assessed the key RuBP (ribulose-1,5-bisphosphate)-generating enzyme activities of fructose 1,6- bisphosphatase (FBPase) and Rubisco. After CS, the FBPase and Rubisco activities of the leaves were decreased to 41% and 32% of the control, respectively. In ME-treated seedlings, the decline in these traits was markedly reduced (22%, and 13%, respectively) (Figure 7A,B).

### 3.7. Starch and Sugar Pool

Compared to the CK group, the cold stress treatment significantly reduced the accumulation of starch, sucrose, soluble sugar, and glucose in pepper seedlings. In ME-treated seedlings, the reduction in the accumulation of sucrose, soluble sugar, and glucose was considerably lower (24%, 23%, 16%, and 22%, respectively) (Figure 7C–F).

### 3.8. Melatonin Biosynthesis Genes

**Figure 8:**
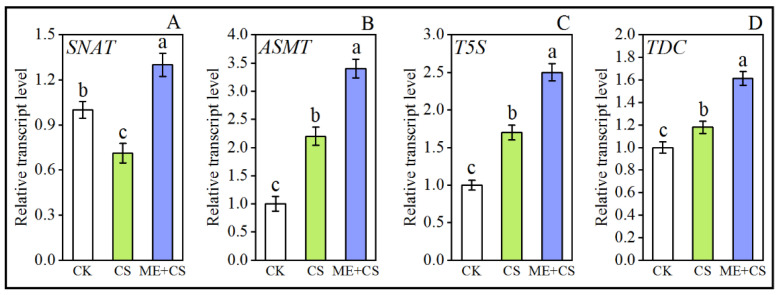
Effects of melatonin treatment on melatonin biosynthesis enzyme-related gene (*SNAT* (**A**), *ASMT* (**B**), *T5S* (**C**), *TDC* (**D**)) expression in pepper seedlings exposed to cold stress. The results are means ± standard errors for n = 9. Significant differences are exhibited by lowercase letters (*p* ≤ 0.05), according to Duncan’s multiple range test.

The core genes that encode crucial enzymes involved in ME biosynthesis are *SNAT*, *ASMT*, *T5S*, and *TDC*. Cold stress considerably enhanced the relative transcription levels of these genes. Additionally, the transcript abundance of these genes further increased in ME-pretreated pepper plants subjected to CS by a considerable amount compared with CK and CS treatments (Figure 8A–D), suggesting that ME biosynthesis may be involved in the mitigation of CS in pepper.

## 4. Discussion

Photosynthesis is a key metabolic process that takes place in plants and is regarded as the core of the living world; furthermore, this process is considered to be very vulnerable to CS [44]. Chlorophyll production is severely hindered or disrupted by CS, which has significant effects on plant growth and productivity. The photosynthetic electron transport system is negatively affected under low-temperature stress, mainly due to elevated membrane viscosities, as well as the restricted diffusion of plastoquinone. In the present study, we confirmed that pretreatment with ME decreased the long-term effects of CS on chlorophyll degradation and carotenoid content inhibition, suggesting that ME might efficiently boost chlorophyll production under stress conditions (Figure 2 and Figure 3). In a recent study, Li et al. [36] observed that ME application efficiently enhanced the chlorophyll pigment molecules and carotenoid content in the leaves of pepper plants under the combined stress of low temperature and low light. Melatonin treatment before CS enhanced the expression of genes involved in ME biosynthesis, which may regulate the optimum chlorophyll content (Figure 8). Previous studies reported that ME supplementation effectively boosts ME biosynthesis gene expression in many plant species under abiotic stress conditions [45,46].

Furthermore, the stomatal state of the leaves affects the activity of photosynthetic processes. Cold-induced stomatal closure may reduce photosynthetic efficiency [6]. The current findings demonstrated that CS caused a reduction in Pn, Gs, Ci, and Tr, but these reductions were significantly reversed by the application of exogenous melatonin (Figure 4). These findings show that the supplementation of exogenous melatonin might alter the opening of stomata in response to CS. Similar results were found in *Arabidopsis thaliana* [47], *Prunus persica* [48], *Citrullus lanatus* [49], and *Solanum lycopersicum* [28]. In a recent study, Altaf et al. [50] reported that exogenous ME application significantly enhanced gas exchange parameters in tomatoes under drought stress. Furthermore, the chloroplast is the plant’s primary site of free radical formation, and requires an effective defense against free radicals and related oxidative damage. Melatonin production in plants may occur in chloroplasts [51].

Chlorophyll fluorescence has become an important technique for studying the photosynthetic parameters of plants under adverse environmental conditions [20]. Significant photoinhibition occurs in the photosystem reaction center under CS conditions. Plants produce excessive energy if the dissipation process stops working, which ultimately leads to a negative impact on the photosystem [44]. The present study revealed that CS dramatically decreased the values of ETR and qP, whereas the values of NPQ and qN were improved (Figure 5). In contrast, ME application efficiently reduced the negative effects of CS by enhancing qP and lowering the NPQ values under CS conditions (Figure 5), suggesting that CS caused significant damage to the photosynthetic apparatus in pepper seedlings, which was then recovered by ME supplementation. The current study also confirms the results of previous studies, which showed that melatonin might protect photosynthetic efficiency [25,52]. Furthermore, the results of the current study are also in line with the existing literature on kiwifruit under drought stress [53], tomatoes under cadmium stress [54], and barley under cold stress [55].

Photosynthetic electron transport is critical for maintaining a precise rhythm for photosynthesis, and providing an optimal flow of energy, which supports healthy plant growth, development, and stress responses [20]. In this study, the photochemistry of PSI and PSII was examined in order to comprehend the detrimental impact of CS on photosynthesis and the potential function of ME (Figure 5). Melatonin may aid in maintaining high photosynthetic efficiency and chlorophyll molecule integrity [53,56]. Our results reveal that Fo was enhanced in CS seedlings, implying that the PSII reaction center was damaged, resulting in a decrease in Fv/Fm, Fv’/Fm’, and фPSII. Conversely, ME application mitigated cold-induced changes to Fo and Fv/Fm (Figure 5). Melatonin positively impacted the quantum yield of PSII and the electron donation to PSII under cold stress [55]. These findings are in line with earlier research on watermelon, peppers, and apples [36,49,57]. Furthermore, ME supplementation efficiently enhanced the photosynthetic capacity of Y(II) under CS conditions (Figure 5). These findings are consistent with previous studies on maize [13], tomatoes [58], and rice [31].

In comparison to PSII, PSI is less susceptible to stress [20]. When seedlings were exposed to stress conditions, a significant increase in P700 oxidation simultaneously reduced the quantum efficiency of PSI (Y(I)), which was associated with an elevation in NPQ, a reduction in the plastoquinone pool, and a decrease in the quantum efficiency of PSII (Y(II)) [59]. On the other hand, the regulation of PSI by melatonin has gained little attention. In this study, we observed that CS dramatically reduced the values of Pm and Y(I). Conversely, ME treatment effectively decreased the adverse effects of CS by increasing Pm and Y(I) values under CS conditions (Figure 5). Similar results were reported for tomatoes, maize, and rice [20,60,61]. In addition, these findings are consistent with reports on several plants that received melatonin pretreatment, which showed lower qN and higher qL levels [53,62]. Furthermore, ME significantly reduced the quantum yield of nonregulated and regulated energy dissipation in PSII, as evidenced by the lower values of Y(NO) and Y(NPQ) in the ME-treated CS plants [20,63]. To further confirm these results, we assessed many PSII reaction center (*CaPSBY* and *CaPSBW*) and PSI reaction center (*CaPSAD*, *CaPSAF*, *CaPSAEA*, *CaPSAH*, and *CaPSAL*)-related genes from the various treatment combinations to examine the possible role of ME in terms of photosynthesis-associated gene expression (Figure 6). Melatonin supplementation enhanced the transcript levels of photosynthesis-related genes in pepper seedling subjected to CS [20].

Cold stress reduces the activity of photosynthesis enzymes such as Rubisco, which is regarded as the most significant photosynthetic enzyme [44]. The present results reveal that CS markedly decreased Rubisco activity; however, ME sustained greater Rubisco activity and enhanced the photosynthetic efficiency under CS conditions (Figure 7). Rubisco is one of the photosynthetic enzymes that contribute significantly to CO_2_ fixation, and is sensitive to low temperatures [64]. Jahan et al. [20] also reported that Rubisco activity was effectively reduced under heat stress. In contrast, ME supplementation efficiently enhanced Rubisco activity under heat stress conditions. Similar results were found in tomatoes and wheat [7,25,29]. In the current study, CS significantly decreased FBPase activity; however, ME-treated CS pepper seedlings displayed greatly enhanced FBPase activity (Figure 7). These results are consistent with previous research on tomatoes [28,65] and kiwifruit [53].

Starch accumulation is strongly related to the activity of photosynthetic apparatus under stressful conditions [66,67]. The current results show that starch and soluble sugar contents in pepper leaves were significantly reduced, but ME application efficiently improved their concentrations under CS conditions (Figure 7). Our findings support those of earlier research, which showed that ME treatment enhanced the accumulation of starch, sucrose, soluble sugar, and glucose in *Solanum lycopersicum* under heat stress conditions [20], *Triticum aestivum* under cold stress conditions [24], *Oryza sative* under salt stress conditions [31], and *Actinidia deliciosa* under drought stress conditions [53]. In conclusion, ME helped to improve pepper seedlings’ CS tolerance, which was reflected by an improvement in sugar metabolism. These results clearly show that exogenous ME supplementation reduced cold-induced photosynthetic limitation, and that ME played a crucial role in ensuring photosynthetic efficiency in stressed situations.

## 5. Conclusions

**Figure 9:**
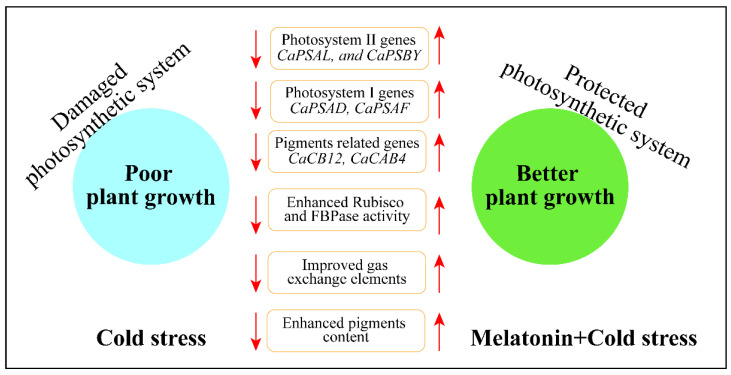
Summary of the mechanism of ME-induced cold stress tolerance in pepper seedlings.

This study investigated the potential function of ME on the photosynthetic performance of pepper seedlings subjected to CS. Figure 9 shows the potential functions of ME and CS treatment on photosynthetic performance. Our findings show that ME application significantly reduced CS-induced photoinhibition by increasing sugar metabolism and upregulating ME biosynthesis genes. Melatonin treatment substantially increased gas exchange parameters and pigment molecules under CS conditions. Furthermore, ME also increased the activity of essential photosynthetic enzymes, such as Rubisco and FBPase. Additionally, ME treatment improved the photochemistry of photosystems II and I, as well as the chlorophyll a fluorescence system. Overall, ME protected pepper seedlings from cold-induced photosynthetic damage by boosting their photosynthetic performance and maintaining the activity of the photosystems.

## Data Availability

Data is contained within the article and Supplementary Material.

## Supplementary Materials

The following supporting information can be downloaded at: https://www.mdpi.com/article/10.3390/antiox11122414/s1.
